# Incorporation of Zinc into Binary SiO_2_-CaO Mesoporous Bioactive Glass Nanoparticles Enhances Anti-Inflammatory and Osteogenic Activities

**DOI:** 10.3390/pharmaceutics13122124

**Published:** 2021-12-09

**Authors:** Haishui Sun, Kai Zheng, Tian Zhou, Aldo R. Boccaccini

**Affiliations:** 1 Department of Oral Maxillofacial-Head and Neck Oncology, Shanghai Ninth People’s Hospital, Shanghai Jiao Tong University School of Medicine, College of Stomatology, Shanghai Jiao Tong University, National Center for Stomatology, National Clinical Research Center for Oral Diseases, Shanghai Key Laboratory of Stomatology, Shanghai 200011, China; haishuisun@126.com; 2 Jiangsu Province Engineering Research Center of Stomatological Translational Medicine, Nanjing Medical University, Nanjing 210029, China; 3 Institute of Biomaterials, University of Erlangen-Nuremberg, 91058 Erlangen, Germany; aldo.boccaccini@ww.uni-erlangen.de

**Keywords:** zinc, bioactive glasses, anti-inflammatory, osteogenic differentiation, immunomodulation

## Abstract

During the healing and repair of bone defects, uncontrolled inflammatory responses can compromise bone regeneration. Biomaterials with anti-inflammatory activity are favorable for bone tissue regeneration processes. In this work, multifunctional Zn-containing mesoporous bioactive glass nanoparticles (Zn-MBGs) exhibiting favorable osteogenic and anti-inflammatory activities were produced employing a sol-gel method. Zn-MBGs exhibited a mesoporous spherical shape and nanoscale particle size (100 ± 20 nm). They were degradable in cell culture medium, and could release Si, Ca, and Zn in a sustained manner. Zn-MBGs also exhibited a concentration-dependent cellular response. The extract of Zn-MBGs obtained by incubation at 0.1 mg/mL (in culture medium) for 24 h could enhance in vitro mineralization, alkaline phosphatase activity, the expression of osteogenesis-related genes, and the production of intracellular protein osteocalcin of rat bone marrow stromal cells (BMSCs). Moreover, the extract of Zn-MBGs at 0.1 mg/mL could significantly downregulate the expression of inflammatory genes and the production of inducible nitric oxide in RAW 264.7 cells, particularly under stimulation of inflammatory signals interferon-γ (IFN-γ) and lipopolysaccharide (LPS). Zn-MBGs also inhibited the pro-inflammatory M1 polarization of RAW264.7 cells induced by LPS and IFN-γ. In summary, we successfully synthesized Zn-MBGs with concentration-dependent osteogenic and anti-inflammatory activities. Zn-MBGs show their great potential in immunomodulation strategies for bone regeneration, representing a multifunctional biomaterial that can be applied to regenerate bone defects under inflammatory conditions.

## 1. Introduction

Repair of bone defects caused by tumor, trauma, or bacterial infection remains a challenging healthcare issue. Bone implants or bone tissue engineering scaffolds (e.g., bioceramics, biodegradable metals) with antibacterial, osteoconductive and osteoinductive properties have been used to repair and regenerate bone defects [1,2,3]. When biomaterials are implanted in vivo, they can trigger immune responses that may compromise or delay bone regeneration. On the other hand, appropriate immune responses can promote bone defect healing and repair [4,5]. Biomaterials able to mitigate undesired inflammatory responses or modulate immune responses are emerging as part of novel strategies to enhance bone tissue regeneration [6,7]. Interactions between biomaterials and immune cells are key in immunomodulatory approaches toward enhanced tissue regeneration [5]. Among various immune cells, macrophages are considered to be a primary immunomodulatory target as their plasticity, polarization, and function can be modulated in response to the environment [8].

Bioactive glasses (BGs) are biologically multifunctional inorganic biomaterials extensively investigated for tissue regeneration applications, given their bone-bonding, pro-osteogenic, and pro-angiogenic abilities [9]. Compared to microsized, irregularly shaped BGs, spherical mesoporous bioactive glass nanoparticles (MBGs) usually possess a larger specific surface area, smaller particle size, and adjustable pore structure, consequently exhibiting more significant bioactivity and enhanced interactions with cells [10]. Given their morphological characteristics, MBGs are also promising carriers of drugs and biologically active ions for various therapeutic strategies (e.g., bone repair, wound healing, cancer treatment) [11]. The immunomodulatory function of BGs is gaining increasing attention as a novel target for bone regeneration [7]. BGs have been shown to exhibit anti-inflammatory activity and the capacity to modulate macrophage responses, including secretion of cytokines and polarization, which results in a microenvironment beneficial for bone regeneration [7]. The immunomodulatory effects of BGs are mainly induced by incorporating biologically active ions, although such effects can also be achieved by loading therapeutic biomolecules.

Bioactive ions have been known for their crucial roles in the regenerative processes of various tissues through specific cellular pathways [12,13]. The role of bioactive ions in immune responses is starting to be understood. Specific bioactive ions have shown anti-inflammatory activity or immunomodulatory effects, consequently enhancing bone regeneration [14,15,16]. For example, the anti-inflammatory effects of cerium, strontium, and boron have been reported based on different cellular mechanisms [7]. Zinc (Zn) ions have also shown anti-inflammatory activity. For example, Zn has been reported to reduce and enhance the secretion of pro-inflammatory and anti-inflammatory cytokines, respectively [17]. In addition, Zn ions have been reported to stimulate osteogenic and antibacterial activities [12,18]. Interestingly, the inclusion of Zn into sulfonated polyetheretherketone could induce a favorable microenvironment regulating macrophage polarization to anti-inflammatory M2 phenotype and stimulating anti-inflammatory and osteogenic cytokine production [19]. 

In our previous study, we have synthesized highly dispersed Zn-containing MBGs (Zn-MBGs) using sol-gel based processing. The inclusion of Zn did not impact the amorphous characteristic and bioactivity of MBGs. Zn-MBGs can sustainably release bioactive ions. In addition, Zn-MBGs were non-cytotoxic against osteoblast-like cells (MG-63). However, in our previous study, the osteogenic and immunomodulatory effects of Zn-MBGs were not investigated. Here, we focused on the interactions of Zn-MBGs with bone marrow stromal cells (BMSCs) and macrophages to understand their osteogenic and immunomodulatory potential, which should lead to expanding the applications of Zn-MBGs in bone tissue regeneration strategies. 

## 2. Materials and Methods

### 2.1. Synthesis and Ion Release Behavior of Zn-MBGs

Zn-MBGs were prepared by employing a microemulsion-assisted sol-gel method as reported in our previous study [20]. Scanning electron microscopy (SEM) (Zeiss Auriga 4750, Jena, Germany) was used to observe the morphology and surface structure of Zn-MBGs and MBGs. The microstructure of Zn-MBGs was investigated by scanning transmission electron microscopy (STEM, Titan3 G2 60–300, FEI, Hillsboro, OR, USA). The particle size of Zn-MBGs and MBGs was determined by analyzing the particles shown in SEM images using Image J analysis software (National Institutes of Health, Bethesda, MD, USA). At least 200 particles were countered to determine the particle size. Ion release profiles of Zn-MBGs and MBGs were evaluated in Dulbecco’s Modified Eagle Medium (DMEM) (HyClone, Logan, UT, USA). In brief, the nanoparticles (5 mg/mL) were soaked in DMEM (supplemented with 10% fetal bovine serum (FBS), 1% penicillin/streptomycin (P/S, GIBCO, Carlsbad, CA, USA)) under 120 rpm agitation at 37 °C for up to seven days. The supernatants were collected at different time points by centrifugation and filtered (0.22 μm). The concentrations of Ca^2+^ and Si^4+^ in the MBGs group and Ca^2+^, Zn^2+^, and Si^4+^ in the Zn-MBGs group were determined using inductively coupled plasma atomic emission spectroscopy (ICP-AES, Agilent 5110, Agilent, Santa Clara, CA, USA).

### 2.2. Preparation of Extracts

The extracts of MBGs and Zn-MBGs were prepared by incubating nanoparticles in DMEM for 24 h at 37 °C at concentrations of 5, 1, and 0.1 mg/mL. The concentrations were selected according to the cytotoxicity results in our previous study [20]. The extracts were collected with centrifugation and filtration for further cell culture studies. The extracts used in the following cell culture experiments were supplemented with 10% FBS and 1% P/S. 

### 2.3. Cell Culture

BMSCs were isolated from femur and tibia of Sprague Dawley (SD) rats aged from three to five weeks, and cultured according to the adherent method [21]. The rats were obtained from the animal center of Shanghai Ninth People’s Hospital, China. The Ethics Committee of the Shanghai Ninth People’s Hospital has approved the animal experiment. BMSCs were then isolated and cultured in DMEM supplemented with 10% FBS, 1% P/S and passaged by trypsinized with 10% trypsin-EDTA (HyClone, Logan, UT, USA). The 2–4 generation BMSCs were used for in vitro experiments. Cell culture medium was changed every three days.

RAW264.7 cell line was purchased from the Chinese Academy of Sciences (Shanghai, China), and cultured and passaged in high glucose DMEM (HyClone, Logan, UT, USA) with 10% FBS, 1% P/S at 37 °C in a humid atmosphere of 5% CO_2_. The cells were subcultured every three days, and the cell culture medium was changed every three days.

### 2.4. Cell Counting Kit-8 (CCK-8) Assay

Effects of Zn-MBGs on the metabolic activity of BMSCs and RAW264.7 cells were tested using the CCK-8 assay. Briefly, BMSCs were seeded in a 96-well plate (Corning Costar Co., New York, NY, USA) at 5 × 10^3^ cells/well. After the cells adhered to the wall for 24 h, the culture medium was replaced by the extracts and cultured for one, three, and seven days. The extracts were changed every three days. Given their rapid proliferation, RAW264.7 cells were cultured at a density of 1 × 10^4^ cells/well with the extracts in 96-well plates for 24 h to evaluate the cytotoxicity of MBGs and Zn-MBGs. At predestined time points, 100 μL fresh medium and 10 μL CCK-8 agent (DOJINDO, Kumamoto, Japan) were used to replace the conditioned medium (extracts). After incubation in darkness for 2 h, the optical density (OD) at the wavelength of 450 nm (OD 450) was measured following the manufacturer’s instructions.

### 2.5. Live/Dead Fluorescence Staining

BMSCs were incubated in 12-well plate (Corning Costar Co., New York, NY, USA) for 24 h at a density of 3 × 10^4^ cells/well. The medium was then changed with different concentrations of extracts and cultured for three and seven days. The extracts were changed every three days. RAW264.7 cells were seeded in 96-well plates at the density of 1 × 10^4^ cells/well. After 24 h, the extracts were added and cultured for a further 24 h. Given the rapid proliferation of RAW264.7 cells, 24 h of culture was performed before the staining, as reported in the literature [22]. At different time points, the cells were washed once using PBS and an appropriate volume of Calcein/Propidium iodide (PI) was added to detect the working fluid (Beyotime, Cat: C2015M, Shanghai, China). After further incubation of 30 min, the samples were photographed. The morphology and viability of the BMSCs and RAW264.7 cells were evaluated and observed with a light microscope (Olympus Corporation, Tokyo, Japan). The percentage of living cells from the live/dead staining of BMSCs and RAW264.7 cells were quantitatively analyzed by Image J software.

### 2.6. Alizarin Red S Staining

BMSCs at a density of 2 × 10^5^ cells/well were plated into 6-well plates and incubated in DMEM with 10% FBS and 1% P/S for 24 h. BMSCs were then treated with extracts supplemented with 0.1 mM dexamethasone, 10 mM β-glycerophosphate, and 50 mM ascorbic acid (Sigma-Aldrich, Hamburg, Germany) for 14 days. The extracts were changed every three days. After culture, the cells were immobilized with 4% paraformaldehyde for 30 min, and 1% ARS (Alizarin Red S) (Shanghai Meiji Biotech Co., Ltd. Shanghai, China) was used to stain the cells following the manufacturer’s instructions before the images were recorded. For the quantitative analysis of ARS staining, the cells were decolorized by 10% cetylpyridinium chloride (Sigma-Aldrich) for 15 min, and the absorbance of the stained cells was recorded at the wavelength of 595 nm (Bio-Tek, Winooski, VT, USA).

### 2.7. Expression of Osteogenesis-Related Genes

The q-PCR technique was used to evaluate the expression of osteogenic differentiation-related genes of BMSCs. BMSCs were seeded into 6-well plates at a density of 2 × 10^5^ cells/well and incubated in DMEM containing 10% FBS and 1% P/S for 24 h. Different concentrations of extracts containing β-phosphoglycerate (10 mM), dexamethasone (0.1 mM), and ascorbic acid (50 mM) were then added and cultured for 7 and 14 days. The extracts were changed every three days. The total RNA was isolated with Trizol reagent (Invitrogen) and reverse transcribed with PrimeScript RT reagent (Takara Bio Inc., Shiga, Japan). The expression of alkaline phosphatase (ALP), RUNX family transcription factor 2 (RUNX-2), collagen I (COL-I), and osteocalcin (OCN) related to osteogenic differentiation was analyzed by Bio-Rad sequence detection system (MYIQ2, Hercules, CA, USA) using real-time PCR (SYBR Premix EX Taq, Takara, Tokyo, Japan). Housekeeper β-actin was used to standardize the results. According to the formula 2^−ΔΔCt^, the data are expressed as the multiple changes of the control. The gene primer sequences mentioned above are shown in Table 1.

### 2.8. Immunostaining of Osteocalcin (OCN)

The expression of osteocalcin (OCN) in BMSCs was detected by immunofluorescence. After 14-day culture with different concentrations of MBGs or Zn-MBGs extracts, BMSCs were immobilized with 4% paraformaldehyde for 30 min, permeated with Triton ×100 (0.5%) for 20 min, and sealed with bovine serum albumin (BSA) for 1 h. The cells were then incubated with an anti-osteocalcin (OCN; Affinity, Cincinnati, OH, USA) (1:100) overnight at 4 °C. The cells were also incubated with Alexa Fluor 555 donkey anti-rabbit antibody (1:200) (Beyotime, Shanghai, China) for 1 h and with DAPI (1:500; Solarbio, Beijing, China) for 5 min before the images were photographed (TE2000-U, Nikon, Tokyo, Japan). The imaging and acquisition parameters were the same for all samples. The fluorescence intensity was acquired from images (×100 times) and quantitatively analyzed by Image J software. 

### 2.9. Expression of Pro-Inflammatory Related Genes

The q-PCR technique was used to evaluate the expression of related genes. RAW264.7 cells were plated into 6-well plates at a density of 3 × 10^5^ cells/well and cultured with DMEM containing 10% FBS and 1% P/S. Different concentrations of extracts were added and cultured for 24 h. The expression of pro-inflammatory related genes interleukin-6 (IL-6), tumor necrosis factor-α (TNF-α), cluster of differentiation 86 (CD86), and interleukin-1β (IL-1β) were evaluated by q-PCR. We also added lipopolysaccharide (LPS) (10 ng/mL) and interferon-γ (IFN-γ) (10 ng/mL) in extracts to induce RAW264.7 cells to differentiate from phenotype M0 to M1 before culture for 24 h to observe the expression of pro-inflammatory-related genes [23]. Total RNA was isolated with TRIzol reagent (Invitrogen) and reverse transcribed with PrimeScript RT reagent (Takara Bio Inc., Shiga, Japan). The expression of pro-inflammatory related genes TNF-α, IL-6, IL-1β, and CD86 was analyzed using a Bio-Rad sequence analysis system (MYIQ2, Hercules, CA, USA) by real-time PCR (SYBR Premix EX Taq, Takara, Tokyo, Japan). GAPDH was used to standardize the results. According to the formula 2^−ΔΔCt^, the data are expressed as the multiple changes of the control. Table 2 shows the forward and reverse primer sequence of the related genes.

### 2.10. iNOS Fluorescence Staining

RAW264.7 cells at a density of 3 × 10^5^ cells/well were plated into 6-well plates. After culture with extracts for 24 h, LPS (10 ng/mL) and IFN-γ (10 ng/mL) were added and cultured for a further 24 h. The cells were immobilized with paraformaldehyde (4%) for 30 min, Triton X-100 (0.5%) for 20 min, and sealed with 0.5% BSA for 1 h. Anti-inducible nitric oxide synthase (iNOS, Affinity, Cincinnati, OH, USA) antibody (1:100) interfered with the cells was cultured overnight at 4 °C. The cells were then incubated with Alexa Fluor 555 donkey anti-rabbit antibody (1:200) (Beyotime, Shanghai, China) for 1 h, and then with DAPI (1:500; Solarbio, Beijing, China) for 5 min. The expression of iNOS was observed by microscope (TE2000-U, Nikon, Tokyo, Japan) after staining. The imaging and acquisition parameters (×100) are the same for all samples. 

### 2.11. Statistical Analysis

All the experiments were performed at least in triplicates. The results were expressed as mean value ± standard deviation (SD). Analysis was conducted using Origin 8.0 (Origin Lab Corporation, Northampton, MA, USA) software. Statistical significance was determined using one-way ANOVA with Dunnett’s multiple comparisons test. Differences between each group and the control group which were statistically significant were indicated by “*”. There were significant differences between different concentrations of MBGs and Zn-MBGs in parentheses, which were denoted by “#”. The significant level was established at * *p* < 0.05. “NS” indicated no significant difference.

## 3. Results and Discussion

### 3.1. Ion Release of Zn-MBGs in Cell Culture Medium

**Figure 1:**
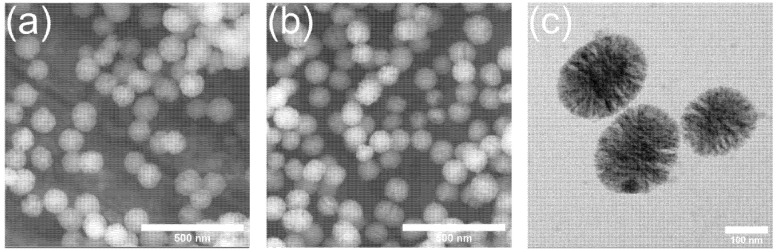
Particle morphology of MBGs (**a**), Zn-MBGs (**b**) in SEM images and Zn-MBGs (**c**) in TEM images.

The microemulsion-assisted sol-gel method was used to synthesize Zn-MBGs and MBGs [20]. Both types of particles exhibited spherical shape and a particle size of 100 ± 20 nm, as shown in Figure 1. Mesopores could be observed in both MBGs and Zn-MBGs. Figure 1c shows TEM image of Zn-MBGs, which clearly indicated the mesopores. The shape and size of the nanoparticles were uniform, which is consistent with the morphology of particles synthesized by the same method [24]. The incorporation of Zn did not show any significant effects on the pore structure of MBGs. The large specific surface area (229 m^2^/g for MBGs and 274 m^2^/g for Zn-MBGs) and mesoporous structure (pore size ~ 3.7 nm) have been confirmed in the previous study [20]. Moreover, the actual compositions of MBGs and Zn-MBGs have been detected to be ~88SiO_2_-12CaO (mol%) and ~84SiO_2_-8CaO-8ZnO (mol%) by ICP-AES [20]. The morphological and compositional characteristics of Zn-MBGs make these particles promising fillers for composites or carriers of biomolecules for various therapeutic applications.

**Figure 2:**
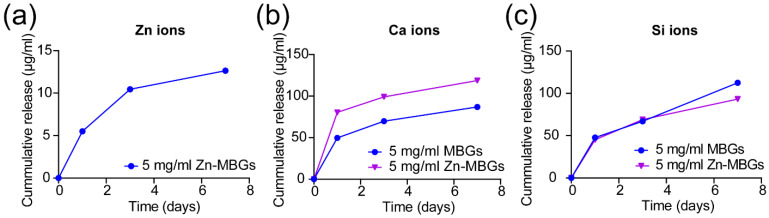
Ion release curves of Zn^2+^ (**a**), Ca^2+^ (**b**) and Si^4+^ (**c**) from Zn-MBGs and MBGs cultured in DMEM at concentration of 5 mg/mL for 1, 3, and 7 days.

We further investigated the ion release of Zn-MBGs and MBGs in cell culture medium. The release of Ca^2+^, Si^4+^, and Zn^2+^ from Zn-MBGs and MBGs after soaking in DMEM for one, three, and seven days is shown in Figure 2. Both MBGs and Zn-MBGs could sustainably release Si^4+^ for up to seven days in DMEM, suggesting the degradation of these silicate nanoparticles. On day seven, MBGs could release a slightly larger amount of Si^4+^ than Zn-MBGs, suggesting the faster degradation of MBGs. Both particles also exhibited a sustained release of Ca^2+^. However, Zn-MBGs appeared to release a larger amount of Ca^2+^ than MBGs. This phenomenon could be attributed to the higher surface reactivity of MBGs that facilitated calcium phosphate precipitation on the particles. The formation of calcium phosphate species could consume Ca^2+^ in solution. Therefore, less Ca^2+^ was detected in the group of MBGs after soaking for seven days. Similar results have been observed in our previous study [20]. Zn-MBGs exhibited a sustained release of Zn^2+^. The released amount of Zn^2+^ from Zn-MBGs was significantly lower than that of Ca^2+^, although the concentrations of CaO and ZnO in Zn-MBGs were comparable (~8.3 mol% CaO and ~7.9 mol% ZnO). This phenomenon has been observed in previous studies [18,23,24], which could be attributed to the more stable Si-O-Zn bonding than Si-O-Ca bonding in Zn-MBGs [25]. However, more investigations are still required to understand the exact degradation and ion release mechanism of Zn-MBGs in vivo, given the complicated surface reactivities (including degradation, mineralization, and protein adsorption) taking place on BGs in vivo [26]. Nevertheless, our results indicated that Zn-MBGs were biodegradable in physiological fluids and could release Ca^2+^, Si^4+^, and Zn^2+^. 

### 3.2. In Vitro Cytotoxicity

**Figure 3:**
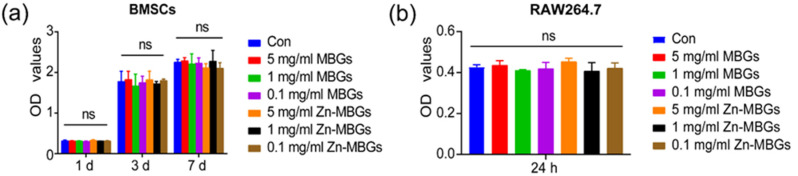
The CCK-8 assay results of BMSCs (**a**) and RAW264.7 cells (**b**) cultured with the extracts of MBGs and Zn-MBGs at different concentrations for one, three, and seven days. NS indicates no significant difference.

We evaluated the cytotoxicity of Zn-MBGs and MBGs (extracts, at 0.1, 1, and 5 mg/mL) against BMSCs and RAW264.7 cells by using the CCK-8 assay. Figure 3a shows no significant difference in OD values between the extract groups (MBGs and Zn-MBGs) and control on all tested concentrations after one-day culture, indicating the non-cytotoxicity of MBGs and Zn-MBGs at all tested concentrations. This result agreed with the reported cytotoxic effects of Zn-MBGs against MG-63 osteoblast-like cells [20]. After culture for three and seven days, no significant reduction in OD values compared to the control in all groups was observed, which indicated that MBGs and Zn-MBGs extracts did not affect the proliferation of BMSCs. We also evaluated the cytotoxicity of the particles against RAW264.7 cells. The result (Figure 3b) showed no significant difference in OD value between experimental groups (regardless of particle concentration) and control after culture for 24 h, indicating the non-cytotoxicity of MBGs and Zn-MBGs against macrophages.

**Figure 4:**
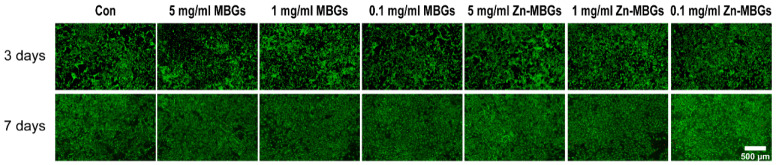
Live/dead staining of BMSCs incubated with MBGs and Zn-MBGs at different concentrations for three and seven days. Green staining represents living cells, while red staining represents dead cells.

The effects of MBGs and Zn-MBGs on cell proliferation were evaluated by living/dead staining. As shown in Figure 4, a large number of living BMSCs (green fluorescence) were observed in both the MBGs and Zn-MBGs groups at all tested concentrations after culture for three and seven days. More living cells could be observed on day seven than day three in all groups, and the cells were densely arranged in multiple layers, indicating the proliferation of BMSCs over time. Quantitative analysis of the percentage of living cells was shown in Supplementary Figure S1. It has been shown that the percentage of living cells in MBGs and Zn-MBGs groups with high concentrations (5 mg/mL) was not significantly different from that of other extracts and the control group. Furthermore, no significant difference in the percentage of living cells could be observed in each group. The results confirmed the non-cytotoxicity of MBGs and Zn-MBGs toward BMSCs, which was consistent with the results of the CCK-8 assay (Figure 3a).

**Figure 5:**
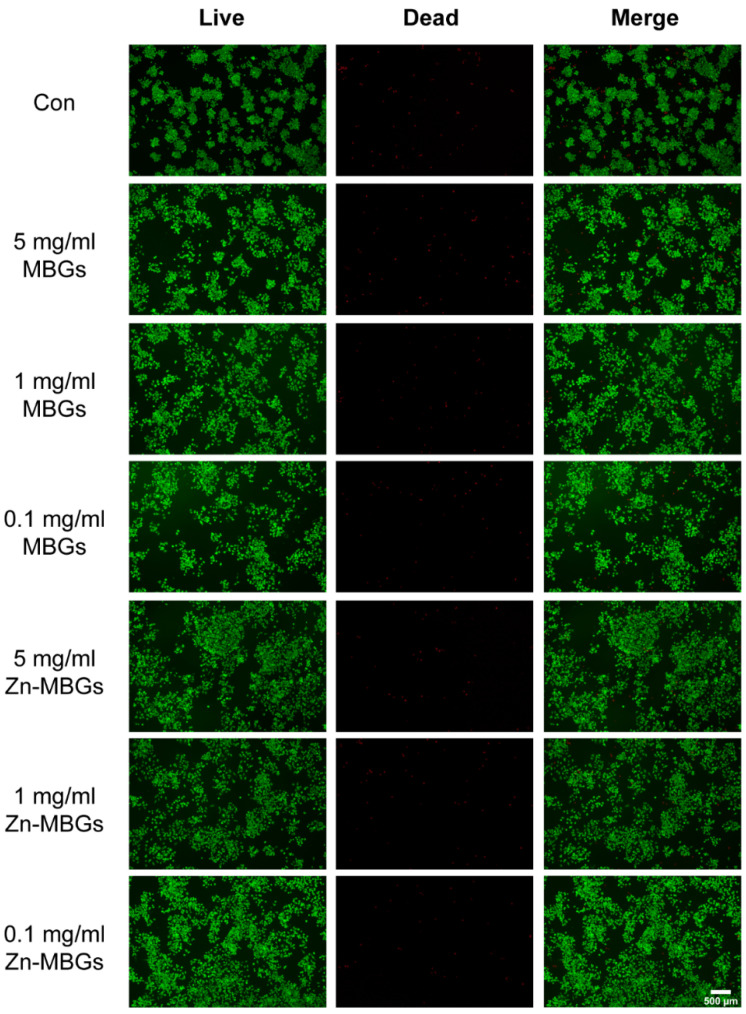
Live/dead staining of RAW264.7 cells incubated with MBGs and Zn-MBGs at different concentrations for 24 h. Green staining represents living cells, while red staining represents dead cells.

Figure 5 shows the live/dead staining results of RAW264.7 cells after culture with extracts of the particles for 24 h. Quantitative analysis of the percentage of living RAW264.7 cells is shown in Supplementary Figure S2. No significant difference among all groups was observed, indicating the non-cytotoxicity of MBGs and Zn-MBGs toward macrophages. The result was consistent with the CCK-8 assay result (Figure 3b).

It is known that both the chemical composition and used concentrations can affect the cytotoxicity of BGs [13]. In our study, an indirect experiment was carried out to evaluate the cytotoxicity of MBGs and Zn-MBGs, given that these bioactive nanoparticles will be mainly used as fillers in polymeric matrices (e.g., hydrogels) to enhance therapeutic actions by releasing ions [27,28], and in these applications, the particles usually are not directly in contact with cells. MBGs have been proved to be non-cytotoxic against various cells (including osteoblasts, fibroblasts) in our previous studies [29,30]. Moreover, incorporation of Zn in MBGs has been shown to be non-cytotoxic against MG-63 cells [20]. Our results showed that Zn-MBGs at the tested concentrations (0.5, 1, and 5 mg/mL) were also non-cytotoxic against BMSCs and macrophages, which suggest that Zn-MBGs could be used in osteoimmunomodulation strategies for bone regeneration [7].

### 3.3. In Vitro Cell Mineralization and Osteogenic Activities

**Figure 6:**
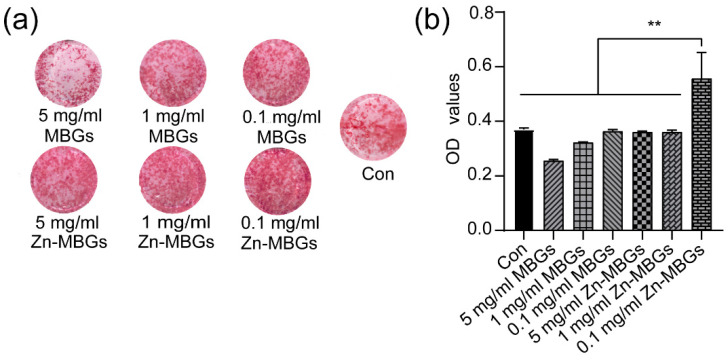
Alizarin red staining (**a**) and its quantitative results (**b**) of BMSCs after culture with MBGs and Zn-MBGs extracts for 14 days. Significant difference between groups with (** *p* < 0.01).

After confirming the non-cytotoxicity of MBGs and Zn-MBGs, we further evaluated the effects of particles on BMSCs mineralization. Figure 6a shows alizarine red staining of BMSCs after culture with extracts for 14 days. 0.1 and 1 mg/mL MBGs induced more calcium nodules than those of the control group and MBGs at 5mg/mL, suggesting a greater osteogenic effect of MBGs at these concentrations. Zn-MBGs induced the deposition of calcium at all tested concentrations. However, 0.1 mg/mL Zn-MBGs led to more calcium nodules than the control and other groups. Figure 6b shows the quantitative results of calcium deposition. Compared to the control, MBGs at a 5 mg/mL concentration induced fewer calcium nodules deposition, while Zn-MBGs at the concertation of 0.1 mg/mL induced more. This phenomenon could be related to concentration-dependent cellular activities of biologically active ions. Although Zn-MBGs at 5 mg/mL could release a larger amount of active ions than those at 0.1 mg/mL, such a relatively high concentration of ions could lead to alkaline extracts that negatively affect cell mineralization and osteogenic activity [31]. Detailed relationship between the concentration of released ions and the osteogenic response of BMSCs will be focused on in future studies.

**Figure 7:**
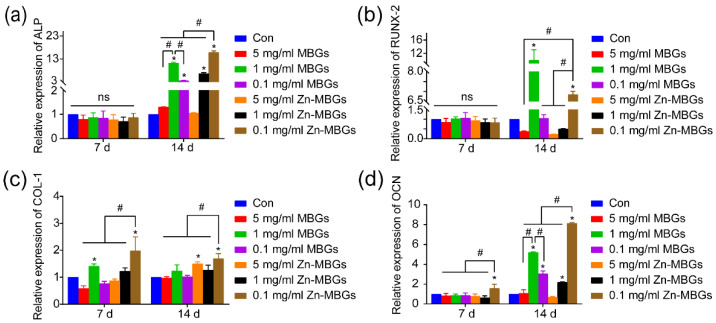
Expression of osteogenic differentiation related genes of BMSCs cultured with different concentrations of MBGs and Zn-MBGs extracts for 7 and 14 days: ALP (**a**), Runx-2 (**b**), Col-1 (**c**), and OCN (**d**). Cells cultured in DMEM were set as the control group. Significant difference between the intervention and control groups (* *p* < 0.05); significant difference between groups (^#^ *p* < 0.05); ns indicates no significant difference.

The osteogenic gene expression in BMSCs after culture with the extracts of particles was investigated using q-PCR technique. Figure 7 shows that no significant difference between ALP and Runx-2 (representative early osteogenic genes) was observed on day seven. On day 14, the expression of ALP in 1mg/mL MBGs was increased compared to the control, indicating the pro-osteogenic activity of MBGs at this concentration. Notably, the 0.1 mg/mL Zn-MBGs group significantly upregulated the expression of ALP and RUNX-2 on day 14, compared to the 0.1 mg/mL MBGs group. However, 1 mg/ ml MBGs group led to significantly greater expression of ALP and RUNX-2 compared to the 1 mg/mL Zn-MBGs group. Notably, 0.1 mg/mL Zn-MBGs induced the most significant expression of ALP on day 14, while 1 mg/mL MBGs led to the most significant expression of RUNX-2 on day 14. The expression of Col-1 and OCN in the 0.1 mg/mL Zn-MBGs group on day 7 and day 14 was greater than the one in other groups. On day 14, MBGs and Zn-MBGs at 1 and 0.1 mg/mL led to greater expression of Col-1 and OCN than the control. The expression of osteogenic genes was consistent with the in vitro cell mineralization results, confirming the favorable osteogenic effects of Zn-MBGs at the concentration of 0.1 mg/mL. These results indicate that MBGs and Zn-MBGs could enhance the osteogenic activity of BMSCs, but these pro-osteogenic activities are concentration-dependent. 

**Figure 8:**
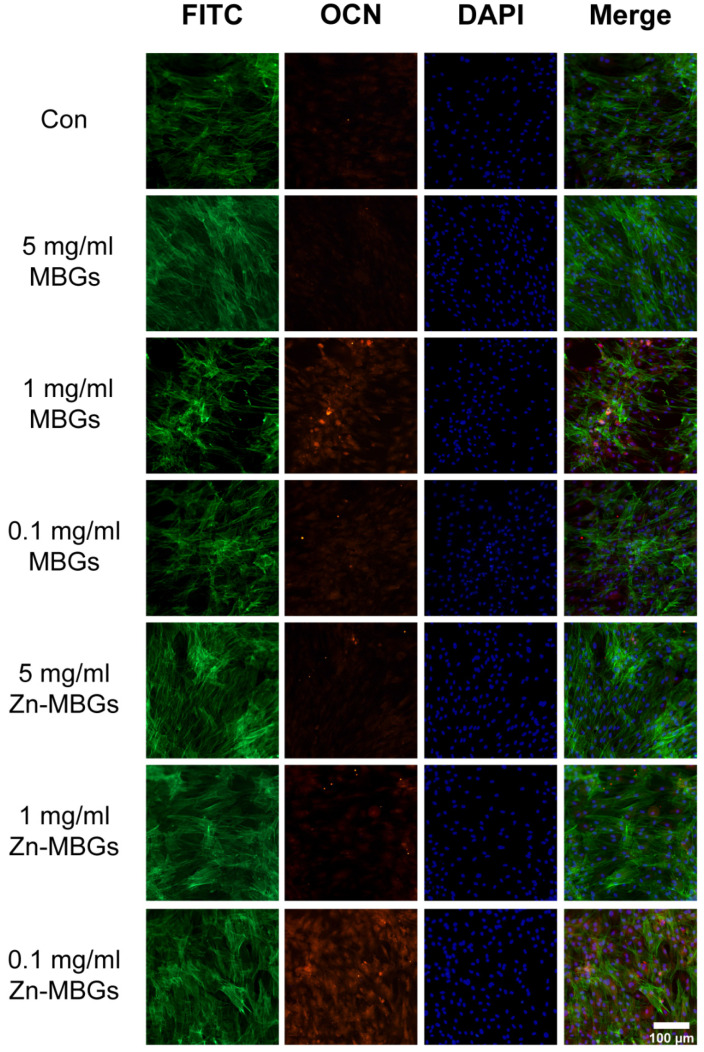
Immunofluorescence staining in BMSCs after culture with the extracts of MBGs and Zn-MBGs for 14 days. Red, green, and blue staining represent OCN, FITC, and DAPI fluorescence staining of BMSCs.

The expression of intracellular protein OCN was also verified by immunofluorescence staining, as OCN is the most stable non-collagenous protein in bone and the determinant of bone formation [32]. As shown in Figure 8, a higher density of BMSCs after culture for 14 days was observed in the group treated with particle extracts, compared to the control. The fluorescence intensity was quantitatively analyzed using Image J, and the results are shown in Supplementary Figure S3. 1 mg/mL MBGs group could express more OCN (red fluorescence) than other MBGs groups, while 0.1 mg/mL Zn-MBGs group could express more OCN than other Zn-MBGs groups, which agreed with the q-PCR results (Figure 7).

It has been reported that Zn could induce intracellular activation of protein-kinase A signaling and promote osteoblastic differentiation of human BMSCs [33]. However, it has also been reported results that Zn-doped BGs did not support osteogenic effects [34]. In addition, Salinas et al. have reported that mesoporous bioactive glass scaffolds incorporated with Zn could promote the colonization of human BMSCs. However, Zn^2+^ only induced significant osteoblast differentiation when combined with osteostatin [35]. These results suggest that the pro-osteogenic effects of Zn-containing BGs are concentration-dependent, probably due to the concentration-dependent therapeutic effects of Zn^2+^. Therefore, a suitable amount of Zn-MBGs should be selected to induce favorable osteogenic effects for bone regeneration applications. Nevertheless, our results indicate that Zn-MBGs could enhance the osteogenic activity of BMSCs. In particular, Zn-MBGs at 0.1 mg/mL could significantly enhance osteogenic activity compared to MBGs and the control. 

### 3.4. Anti-Inflammatory Effects of Zn-MBGs

**Figure 9:**
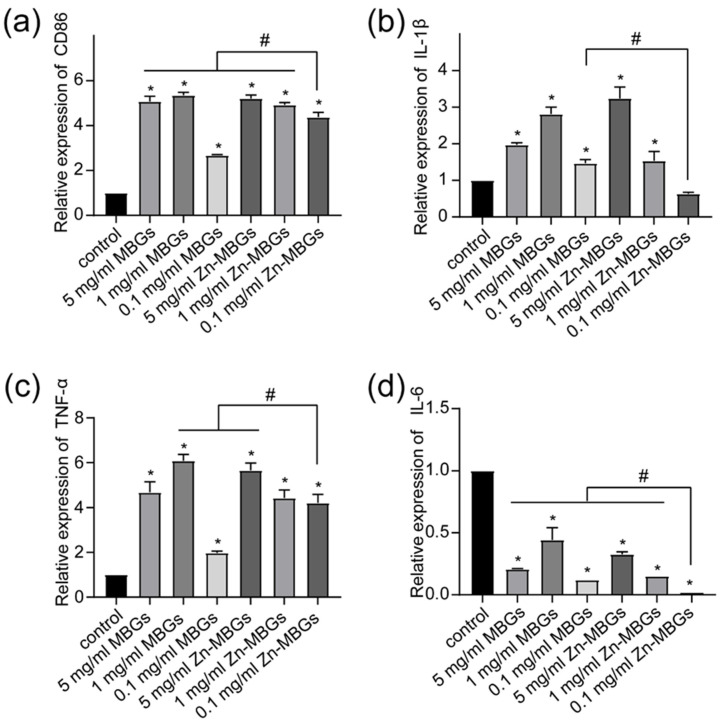
The relative mRNA expression levels of RAW264.7 inflammatory cytokines: CD86 (**a**), IL-1 β (**b**), TNF-α (**c**), IL-6 (**d**) mRNA by MBGs and Zn-MBGs. Significant difference between the intervention and control groups with * *p* < 0.05; significant difference between groups with ^#^ *p* < 0.05.

RAW264.7 cells have prominent macrophage characteristics and can be induced to different cell phenotypes by various stimuli. To evaluate the effect of Zn-MBGs on macrophage polarization, we treated RAW264.7 cells using different concentrations of particle extracts for 24 h and examined the phenotypes and the expression of inflammatory factors. As shown in Figure 9, compared to the control, cell membrane protein CD86 (characteristic M1 macrophage marker) was significantly upregulated by extracts regardless of concentrations, indicating that the presence of MBGs and Zn-MBGs could induce macrophage to pro-inflammatory phenotype M1. The pro-inflammatory mediators IL-1β and TNF-α were synchronously upregulated, indicating the occurrence of inflammatory responses induced by MBGs and Zn-MBGs. However, the expression of the pro-inflammatory gene IL-6 was downregulated in the presence of MBGs and Zn-MBGs (Figure 9d). Notably, 0.1 mg/mL Zn-MBGs could downregulate the expression of IL-1β, as compared to the control (Figure 9b). Moreover, at relatively low concentrations of particles (1 and 0.1 mg/mL), Zn-MBGs could downregulate the expression of pro-inflammatory IL-6 and IL-1β in comparison to MBGs; however, CD86 and TNF-α were upregulated by Zn-MBGs compared to MBGs.

**Figure 10:**
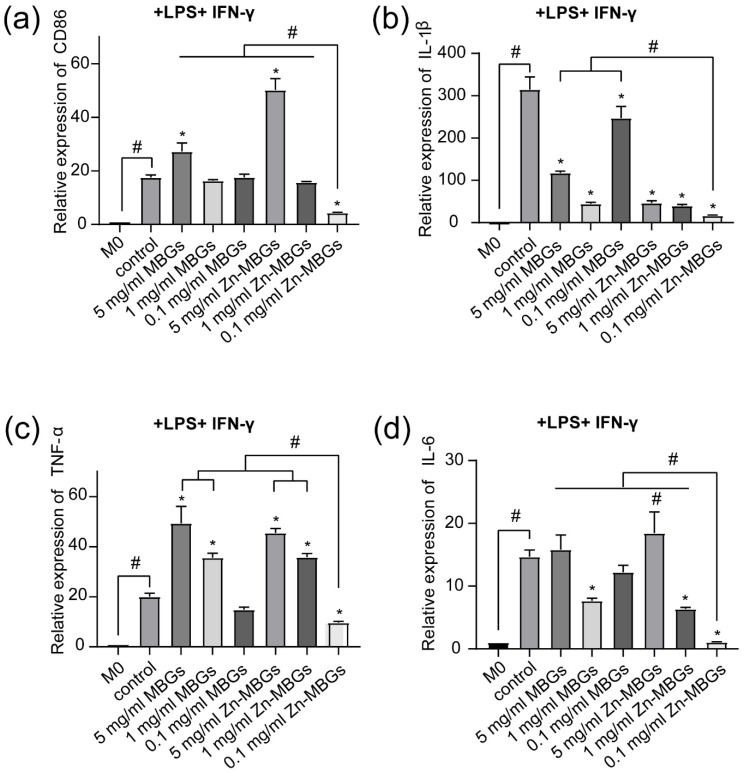
The relative mRNA expression levels of inflammatory cytokines in RAW264.7 after culture with MBGs and Zn-MBGs in the presence of LPS and IFN-γ for 24 h: CD86 (**a**), IL-1β (**b**), TNF-α (**c**), IL-6 (**d**). M0 was the blank control group, and the Control group was in the M1 state after adding stimulus. Significant difference between the intervention and the control group with * *p* < 0.05; significant difference between groups with ^#^ *p* < 0.05.

The inflammatory model of RAW264.7 cells induced by IFN-γ (10 ng/mL) and LPS (10 ng/mL) is a classic in vitro inflammatory model [23,36]. In order to further verify the anti-inflammatory effects of Zn-MBGs, we stimulated RAW264.7 cells with extracts and LPS and IFN-γ for 24 h, and detected the expression of inflammatory genes. A significant increase in the expression of CD86 in the control (Con) compared to the unstimulated group (M0) was observed (Figure 10), which indicates that macrophages (stimulated by LPS and IFN-γ) were converted to pro-inflammatory phenotype M1. Notably, Zn-MBGs at the concentration of 0.1mg/mL significantly downregulated CD86 expression compared to the control (Figure 10a), which indicates that Zn-MBGs at this concentration could inhibit the polarization of macrophages to M1. In the attendance of LPS and IFN-γ, the expression of pro-inflammatory genes IL-1β, TNF-α, and IL-6 in the control group was significantly upregulated compared to the group without LPS and IFN-γ stimulation (M0). The expression trend of IL-1β, TNF-α, and IL-6 was similar to that of CD86. In comparison to the control group, Zn-MBGs at 0.1 mg/mL significantly decreased the expression of IL-1β, TNF-α, and IL-6, indicating the anti-inflammatory effects of Zn-MBGs in the presence of LPS and IFN-γ. Notably, the incorporation of Zn into MBGs could effectively reduce the expression of pro-inflammatory genes IL-1β, TNF-α, and IL-6, which was similar to the observation in the experiments without LPS and IFN-γ stimulation (Figure 9). Taken together, our results indicate that Zn-MBGs, at a suitable concentration (0.1 mg/mL in this case), exhibit effective anti-inflammatory activity under the stimulation of pro-inflammatory stimuli LPS and IFN-γ. 

**Figure 11:**
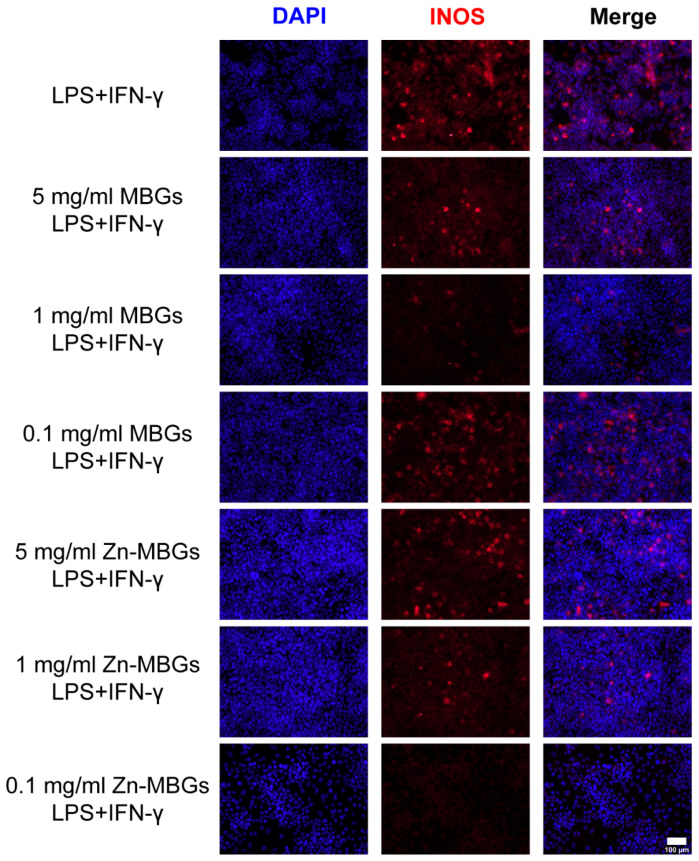
iNOS fluorescence staining of RAW264.7 cells in the presence of LPS (10 ng/mL) and IFN-γ (10 ng/mL) after culture with extracts of MBGs and Zn-MBGs for 24 h. Red and blue staining represent INOS and DAPI, respectively.

Inducible nitric oxide (iNOS) is a major rate-limiting enzyme in an inflammatory response. To further verify the anti-inflammatory effect of Zn-MBGs, an iNOS immunofluorescence experiment was carried out. Figure 11 shows INOS immunofluorescence of RAW264.7 after 24 h culture with particle extracts in the presence of LPS and IFN-γ. As can be seen, the group without extracts exhibited the strongest red fluorescence (expression of INOS) among all groups, indicating the induction of inflammation to the greatest extent. Zn-MBGs at 0.1 mg/mL exhibited the lowest induction of INOS in all groups, as indicated by the weakest red fluorescence, which confirmed the significant anti-inflammatory effects of Zn-MBGs at 0.1 mg/mL. The expression of inflammation gene mRNA showed the same trend as that of INOS staining (Figure 10). 

Previous studies have reported that Zn could induce monocytes to differentiate into macrophages, but the polarization direction of macrophages depends on the concentration of Zn [37]. For example, Zn^2+^ at 100 × 10^−6^ M could upregulate the pro-inflammatory cytokines (such as IL-1β, IL-6, and TNF-α) release. In contrast, Zn^2+^ at a lower concentration of 1.25 × 10^−6^ M could reduce pro-inflammatory cytokine expression [19]. In our study, Zn-MBGs at 0.1 mg/mL clearly showed anti-inflammatory effects as indicated by the downregulated anti-inflammatory gene expression in the presence of stimuli LPS and IFN-γ, which was probably due to the amount of released Zn ions favorable for anti-inflammatory effects. It is worth mentioning that the expression of IL-1β and IL-6 in the 1 mg/mL MBGs group was also significantly downregulated, compared to that in the control group. However, the extent of downregulated expression was lower than that in the 0.1 mg/mL Zn-MBGs group. It has been reported that binary SiO_2_-CaO MBGs could also induce anti-inflammatory effects at suitable concentrations [30,38], probably due to the synergistic effects of Si^4+^ and Ca^2+^. It has been reported that Zn plays an anti-inflammatory role mainly by negatively regulating NF-κB [39]. NF-κB is a crucial regulator of the expression of pro-inflammatory cytokines and other signaling molecules [40,41]. A variety of extracellular signals (such as LPS and IFN-γ) can activate IκB kinase complex (IKK) that induces gene transcription of a variety of inflammatory cytokines [42]. It is known that the key Zn transporter ZIP8 can be upregulated in response to infection or cytokines, inhibiting the phosphorylation of IkB, and subsequently blocking the activation of NF-κB [40,43]. The released Zn from BGs could be beneficial for the activity of Zn transporter ZIP8, thus inhibiting NF-κB and inducing anti-inflammatory activity. Zn-mediated inhibition of NF-κB can also be induced through the inhibition of LPS-induced activation of NF-κB and IKKb [43], which could explain the greater anti-inflammatory effects of Zn-MBGs in the presence of pro-inflammatory stimuli. However, more experimental results are still required to support this hypothesis, which will be a focus in our future studies.

Macrophages can switch phenotypes in response to the local microenvironment [44]. They usually exhibit pro-inflammatory (M1) and anti-inflammatory (M2) phenotypes [45]. A well-regulated and timely phenotype switch from M1 to M2 is beneficial for bone tissue regeneration. Numerous strategies (e.g., morphology optimization, incorporation of active ions) can be used to modify biomaterials to control macrophage behavior toward enhanced bone regeneration. Recently, the roles of bioactive ions in the recruitment, maturation, and activation of macrophages and the secretion of pro-inflammatory and anti-inflammatory cytokines are attracting increasing attention due to their potential in immunomodulation toward tissue regeneration [7]. Given the anti-inflammatory activity and the potential of macrophage polarization modulation and ability to enhance osteogenesis of BMSCs, Zn-MBGs are expected to be effective immunomodulatory biomaterials for tissue regeneration. The immunomodulation effects of Zn-MBGs will be focused on in our future studies.

## 4. Conclusions

Zn-MBGs were successfully produced by using a sol-gel method. Zn-MBGs exhibited a spherical shape and a particle size of approximately 100 nm. Zn-MBGs were biodegradable in physiological fluids and could release biologically active Si, Ca, and Zn ions sustainably. Zn-MBGs exhibited concentration-dependent osteogenic and anti-inflammatory activities. At 0.1 mg/mL, Zn-MBGs could stimulate calcium deposition and ALP activity of BMSCs and upregulate the expression of osteogenesis-related genes ALP, Runx-2, Col-1, and OCN. Zn-MBGs also showed significant anti-inflammatory activity at 0.1 mg/mL, particularly in the presence of inflammatory signals LPS and IFN-γ. Moreover, Zn-MBGs could inhibit the polarization of macrophages to pro-inflammatory phenotype M1 under the stimulation of LPS and IFN-γ. Our preliminary results indicate that Zn-MBGs have favorable in vitro osteogenesis and anti-inflammatory activities under inflammatory conditions, which are potentially beneficial for bone tissue regeneration applications.

## Figures and Tables

**Table 1:** The primers used for the evaluation of osteogenesis-related gene expression in BMSCs.

Genes	Forward Primer Sequence	Reverse Primer Sequence
ALP	ATGCTCAGGACAGGATCAAA	CGGGACATAAGCGAGTTTCT
RUNX2	GGGACTGGTACTCGGACAAT	GGCCTTCTCATCCAGTTCAT
COL-I	AGCTCGATACACAATGGCCT	CCTATGACTTCTGCGTCTGG
OCN	CAGACAAGTCCCACACAGCA	CCAGCAGAGTGAGCAGAGAG
β-actin	CCTCTATGACAACACAGT	AGCCACCAATCCACACAG

**Table 2:** The primers used for the evaluation of pro-inflammatory-related gene expression in RAW264.7 cells.

Genes	Forward Primer Sequence	Reverse Primer Sequence
TNF-α	GCCTATGTCTCAGCCTCTT	GGTTGACTTTCTCCTGGTAT
IL-6	CGATAGTCAATTCCAGAAACCGC	TTGGGAGTGGTATCCTCTGTGAAG
IL-1β	GCAACTGTTCCTGAACTCAACT	ATCTTTTGGGGTCCGTCAACT
CD86	TGTTTCCGTGGAGACGCAAG	TTGAGCCTTTGTAAATGGGCA
GAPDH	GGACACTGAGCAAGAGAGGC	TTATGGGGGTCTGGGATGGA

## Data Availability

The data used to support the findings of this study are available from the corresponding author on request.

## Supplementary Materials

The following are available online at https://www.mdpi.com/article/10.3390/pharmaceutics13122124/s1, Figure S1: Concentrations of living cells in the live/dead staining of BMSCs after culture with the extracts for 3 and 7 days. Figure S2: Concentrations of living cells from the live/dead staining of RAW 264.7 cells after culture with the extracts for 24 h. Figure S3: Quantitative analysis of OCN expression in BMSCs after culture with the extracts of MBGs and Zn-MBGs for 14 days.
