# Supplementary material for: Incorporation of Zinc into Binary SiO2-CaO Mesoporous Bioactive Glass Nanoparticles Enhances Anti-Inflammatory and Osteogenic Activities
Source: Pharmaceutics. 2021 Dec 9;13(12):2124. doi: 10.3390/pharmaceutics13122124 (PMC8705893; doi:10.3390/pharmaceutics13122124)
Supplement: Supplementary file 1 [file pharmaceutics-13-02124-s001.zip › pharmaceutics-1458299-supplementary.pdf]

pharmaceutics

Supplementary Materials: Incorporation of Zinc into Binary SiO_2-CaO Mesoporous Bioactive Glass Nanoparticles Enhances Anti-Inflammatory and Osteogenic Activities

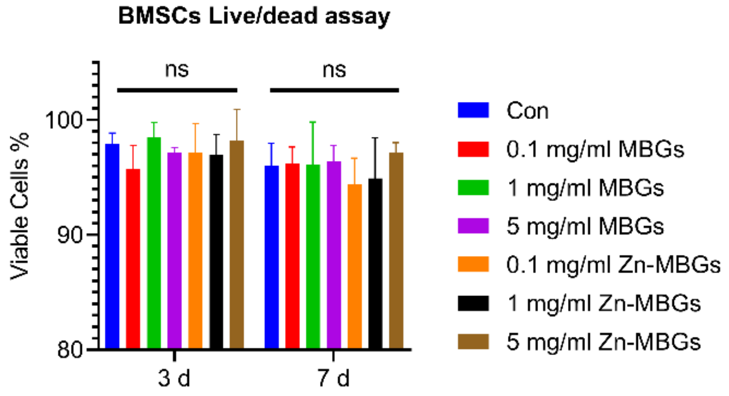

Figure S1. Concentrations of living cells in the live/dead staining of BMSCs after culture with the extracts for 3 and 7 days.

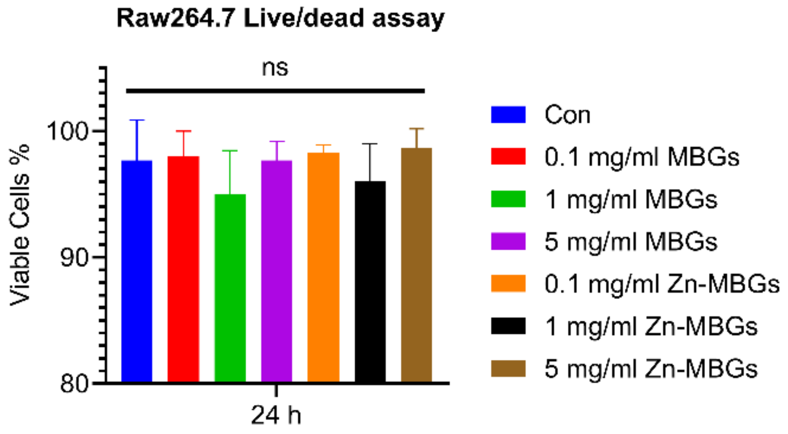

Figure S2. Concentrations of living cells from the live/dead staining of RAW 264.7 cells after culture with the extracts for 24 h

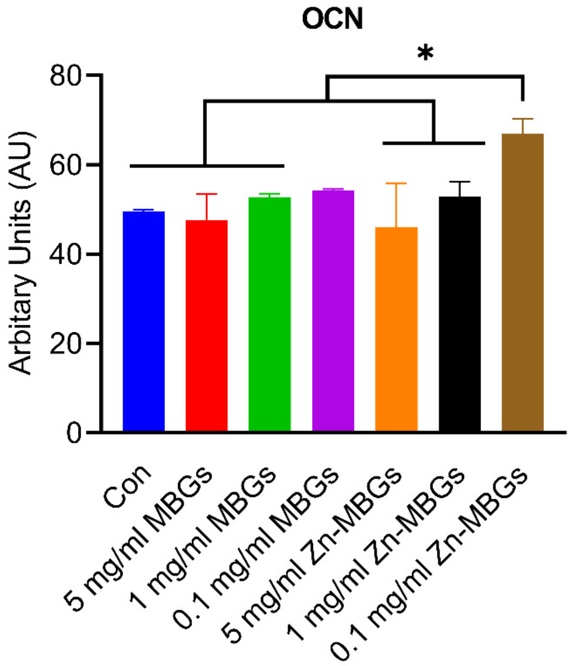

Figure S3. Quantitative analysis of OCN expression in BMSCs after culture with the extracts of MBGs and Zn-MBGs for 14 days.
